# News and Notes

**Published:** 1901-01

**Authors:** 


### *NEWS AND NOTES.*

---

DR. JOHN B. MURPHY has been elected to the professorship in surgery in the Northwestern University Medical School.

---

THE ninety-fifth annual session of the Medical Society of the State of New York will be held in Albany, January 29, 30, 31, 1901.

---

NEW BUILDINGS FOR HARVARD MEDICAL SCHOOL.—Harvard Medical School having outgrown its accommodations, is to have new buildings on the old Francis estate at Brookline.

---

MISSISSIPPI VALLEY MEDICAL ASSOCIATION.—The next meeting will be held at Put-in-Bay, O., September 10–12, 1901, under the presidency of Dr. A. H. Cordier, of Kansas City, Mo.

---

"DIVINE DOWIE" attempted to outwit the United States authorities by sending some lace-workers across from England as first-class passengers. They were not permitted to land.

---

ARMY SURGEONS WANTED.—In his recent report Surgeon-General Sternberg says that there is a demand for a permanent increase in the number of regularly commissioned surgeons.

---

SEVENTY-ONE per cent. of the medical freshmen in the University of Pennsylvania have been matriculates at some college or university. In the law department fifty-seven per cent. are college men.

---

IN appendicitis, even though the temperature be normal or only slightly elevated, it is generally conceded that an operation is indicated when the combination of a rapid pulse and respiration exists.—*Medical Record.*

JOHN CONTEE FAIRFAX, M.D., the only American physician who ever bore a British title of nobility, died recently at his home in Virginia. He was the eleventh Baron Fairfax of Cameron, in the peerage of Scotland—*Medical Times*.

---

A RIOT occurred at Omaha Medical School on November 15. Two students were seriously injured; one may die. The fight between medical and dental students began with fists. A railing was demolished and the balusters used as weapons.

---

AN item from Chicago says that the opening of the great drainage canal has given every city along the Illinois river and the Mississippi as far as St. Louis purer water than any of them ever had before. And yet Washington growls about Cumberland.

---

DR. R. A. WITTHAUS, of New York, will have to sue for his bill for examining the stomachs of Mrs. Catherine J. Adams and Henry C. Barnett, which he made on the direction of the district attorney in the Molineaux case. His bill amounts to $18,550.

---

IN Cementon, Pa., out of 200 families 118 have typhoid fever. In twenty-two families every member is said to be down with the disease. It is doubtful if any town in the country could show such a great proportion of susceptible individuals as these figures would indicate.

---

As a result of eating canned deviled crabs twenty guests at a silver wedding celebration in Gainesville, Ga., were made seriously ill. Four out of the number have died, including the hostess. It is said that the cans were opened on Saturday, but the contents were not eaten until the following Tuesday.

---

CHRISTIAN scientists of Baltimore, says an exchange, are scandalized over the action of one of their leading healers, who is just recovering from a severe illness. The young woman who has brought reproach upon the cause did it not only by getting sick like ordinary mortals, but by calling in a physician as other human beings do.

---

DR. H. A. WEST, of Galveston, has been appointed secretary of the section in medical jurisprudence to represent the United States at the forthcoming meeting of the Pan-American Medical Congress, which meets in Havana, Cuba, December 26 to 29. Dr. West is also member of the auxiliary committee and will be pleased to receive titles for papers in any of the sections.

---

ONE method by which infectious disease is distributed about a city was recently discovered in East Baltimore, Md. A young child died of diphtheria, and the mother, having no mourning clothes of her own, borrowed some from a friend. After the funeral the funeral garments were returned. A day or so afterward diphtheria developed in the family of the owner of the clothes.

---

ONE of the most unique announcements in Polk's Medical Directory for 1900 is that of Dr. Joseph Marriott of Murray, Utah, who gravely announces: "My specialty is diphtheria, erysipelas, cramps, colic, bruises and the fevers." Verily, a new specialty is born every day. Another specialty of interest is that of Dr. Elzie Emmett Grey, of Indianapolis, who declares that her "practice is limited to diseases of cancer."

---

IN a recent lecture delivered in Paris, Dr. Pozzi paid a glowing tribute to American surgery, characterizing the surgeons of the United States as "scientifically audacious and brilliantly cool." He declared that many important surgical improvements were solely due to American discoverers, and made the startling statement that if the American treatment for appendicitis had been better known, Gambetta's life could have been saved. Pozzi is well known in the United States, which country he visited a few years ago.

A COMPOSITE PROFESSION.—Dr. Adam H. Wright, in his President's address before the Ontario Medical Association, calls attention to the following advertisement, taken from a newspaper of Shakespeare's time:

> WANTED—In a family who have had bad health, a sober, steady person in the capacity of doctor, surgeon and man-midwife. He must occasionally act as butler and dress hair and wigs. He will be required sometimes to read prayers, and preach a sermon every Sunday. A good salary will be given.

---

WE have received an official communication that in consequence of the yellow fever which has been existing in Havana during the past two months, although the epidemic has now nearly subsided, it has been deemed prudent to postpone the meeting of the Pan-American Medical Congress till February. Due notice of the date will be published. Judging from the large number of letters received desiring postponement, in consequence of the meeting being set for the Christmas holidays, it is believed that this change will not occasion wide inconvenience.

---

DR. J. C. CULBERTSON, editor of the *Cincinnati Lancet-Clinic*, gave an interesting "evening of authors" at the Hotel Sterling to meet Rev. and Mrs. Henry C. Culbertson, at which the leading literary lights of the Ohio Valley were present, including Dr. Charles Frederick Goss, author of "The Redemption of David Corson"; Dr. John Uri Lloyd, author of "Stringtown on the Pike"; Coates Kinney, author of "Rain on the Roof"; John J. Piatt, Dr. W. H. Venable and some forty others. A unique feature was the dining table set in the form of a hollow square, in the center of which was another table piled with copies of the books written by the guests of the evening.

---

CHOLERA has, according to the *Philadelphia Medical Journal*, always been exceedingly virulent and fatal among Indian coolies employed by tea-planters, especially those who labor on shipboard. At first they would not submit to treatment, but the advantages of inoculation have become so apparent that they now seek it of their own accord, and the tea-planters are inserting special clauses in their contracts calling for inoculated coolies in preference to any others. In some of the tea districts, in which the mortality used to be very high, cholera has practically been exterminated. Notes on a series of experiments, carried on in Calcutta, show that the results, considered as a whole, were as follows: 654 uninoculated, 71 deaths; 402 inoculated, 12 deaths. Proportion, 363 to 1; reduction of mortality by 72.47 per cent.

---

THE *Philadelphia Medical Journal* is authority for the following: At Odessa the liman cure is becoming popular. The limans are sheets of water that have been isolated from the sea and converted into salt lakes. The waters are concentrated by evaporation and are thought to possess great therapeutic value. The principal salts contained in them are the chlorides of sodium, potassium and magnesium; calcium sulphate and magnesium, or sodium bromide. A black, slimy substance of animal and vegetable composition covers the bottoms of the lakes, which contains iodine, bromine, sulphur, sulphuretted hydrogen, and oleic and valerianic acids. Patients bathe either in the open lakes or in baths with the water at different degrees of concentration and temperature. The diseases treated are rickets, scrofula, chronic rheumatic affections, and certain chronic skin diseases.

---

MANY a good story, according to *Health*, is told of Mr. Treves among those who know him best. Here is a sample from our contemporary, the *Leisure Hour*. He was traveling on one occasion by sea with a friend, and the latter having a bad foot requiring slight surgical treatment, Mr. Treves took him along to the ship's doctor, who soon made preparation to operate. A mild suggestion from Mr. Treves as to using a grooved needle was not received with favor, the doctor preferring a knife about a foot long. At a second even milder and more deferential suggestion that carbolic was a rather good thing sometimes to use, the great surgeon got such an indignant look that he modestly retired. The doctor then made some cutting remarks about "amateurs," and asked the name of the vanquished one. "Oh, his name is Treves," replied the patient quietly. "Any relation to the man who wrote those books?"—pointing to a complete row of Mr. Treves's works. "Happens to be the same man," was the smiling reply.

